# Optimization of Tibial Stem Geometry in Total Knee Arthroplasty Using Design of Experiments: A Finite Element Analysis

**DOI:** 10.3390/bioengineering12020172

**Published:** 2025-02-11

**Authors:** Hyun Hee Lee, Hyoung-Taek Hong, Jong-Keun Kim, Yong-Gon Koh, Kwan Kyu Park, Kyoung-Tak Kang

**Affiliations:** 1 Department of Orthopaedic Surgery, International St. Mary’s Hospital, Incheon 22711, Republic of Korea; emprany@ish.ac.kr; 2 Skyve R&D LAB, Seoul 07217, Republic of Korea; 3 Department of Orthopaedic Surgery, Heung-K Hospital, Siheung 14999, Republic of Korea; 4 Joint Reconstruction Center, Department of Orthopaedic Surgery, Yonsei Sarang Hospital, Seoul 06702, Republic of Korea; 5 Department of Orthopedic Surgery, Severance Hospital, Yonsei University College of Medicine, Seoul 03722, Republic of Korea; 6 Department of Mechanical Engineering, Yonsei University, Seoul 03722, Republic of Korea

**Keywords:** tibial stem geometry, design of experiments, finite element analysis

## Abstract

The stability of the tibial component in Total Knee Arthroplasty (TKA) is critical to preventing aseptic loosening, a major cause of implant failure. However, existing tibial stem designs often lead to stress shielding and bone resorption, highlighting the need for further optimization. This study addresses these challenges by employing the Design of Experiments (DOE) methodology, specifically utilizing a full factorial design approach combined with finite element analysis (FEA), to optimize the geometry of the tibial stem. The material properties of the cortical and cancellous bone, as well as the tibial tray, were assigned based on values from the literature, representing their elastic moduli and Poisson’s ratios. For boundary conditions, the distal end of the tibia was fully constrained to simulate realistic load transfer, while compressive loads representative of walking and daily activities were applied to the tibial base. Key design parameters, including stem diameter, length, mediolateral ratio (M/L ratio), and wing angle, were systematically analyzed. The results identified stem diameter and length as the most influential factors in improving biomechanical performance, while the wing angle showed minimal impact. The optimized design, featuring a stem diameter of 12 mm, length of 40 mm, M/L ratio of 0.61, and a wing angle of 60°, demonstrated significant reductions in stress shielding and aseptic loosening compared to conventional models. These findings provide valuable insights into enhancing the long-term success of TKA implants by balancing implant stability and minimizing bone resection.

## 1. Introduction

Total Knee Arthroplasty (TKA) is one of the most widely performed surgical procedures, primarily aimed at alleviating pain and restoring functionality in patients suffering from severe knee joint disorders such as osteoarthritis [1]. Despite its clinical success, the long-term outcomes of TKA are heavily dependent on the stability and durability of the tibial component, especially the tibial stem [2]. The tibial stem plays a critical role in enhancing the fixation of the tibial baseplate to the bone, thereby reducing the risk of aseptic loosening—a leading cause of implant failure [3]. However, clinical cases have reported complications such as non-uniform stress distribution, malalignment, malunion, and instability, which highlight the need for improved designs that minimize such failures while enhancing long-term patient outcomes.

Various tibial stem designs have been employed to address the challenges associated with TKA, with the two most common being the “cross-shaped” and “cylinder with wing” designs. While these designs have been successful in improving implant stability, there remains a significant need to optimize these designs further to minimize stress shielding and bone resorption while ensuring the long-term stability of the implant. In particular, the “cylinder with wing” design has gained widespread clinical use due to its balance of mechanical stability and ease of implantation. The biomechanical implications of design parameters such as stem length, diameter, wing angle, and anterior wing inclusion have not been thoroughly investigated, leading to gaps in understanding their influence on clinical outcomes.

Previous studies have explored various tibial stem designs and their effects on implant performance; however, several limitations remain. Many studies have relied on simplified models that fail to capture the complexities of actual clinical scenarios, such as variability in patient anatomy, implant alignment, and loading conditions [4,5]. Additionally, these studies have not sufficiently addressed the trade-offs between minimizing stress shielding and reducing the risk of aseptic loosening, leaving a significant research gap in optimizing tibial stem designs.

In this study, we employ DOE methodology to systematically investigate the effects of key design parameters on the biomechanical performance of the tibial stem in TKA. The parameters analyzed include stem length, stem diameter, wing angle, and the addition of an anterior wing, which is a novel design feature inspired by the cross-shaped design. By conducting a series of simulations and analyses, we aim to identify an optimized tibial stem design that balances mechanical stability, stress distribution, and bone preservation.

The findings from this research are expected to contribute to the development of more effective tibial components for TKA, potentially improving patient outcomes and extending the lifespan of the prosthesis. Ultimately, the optimized tibial stem design derived from this study could lead to a significant advancement in the field of orthopedic implant design. By advancing the understanding of tibial stem design, this study has the potential to contribute significantly to the field of orthopedic implant design and TKA outcomes.

## 2. Materials and Methods

### 2.1. Conventional Design Analysis

**Figure 1:**
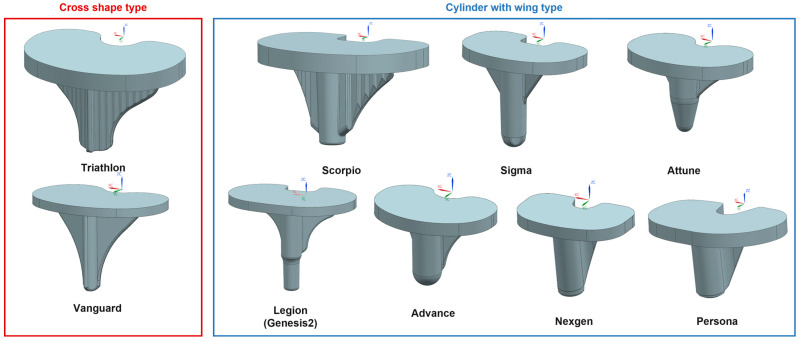
Types of stem design: Cross shape type versus Cylinder with wing type.

There were two common types of stem design which are ‘Cross shape’ and ‘Cylinder with wing’ type among conventional TKA (Figure 1). In this study, ‘Cylinder with wing’ design, more commonly used in the surgery, was selected. Also, we apply anterior wing concept from cross shape type.

**Figure 2:**
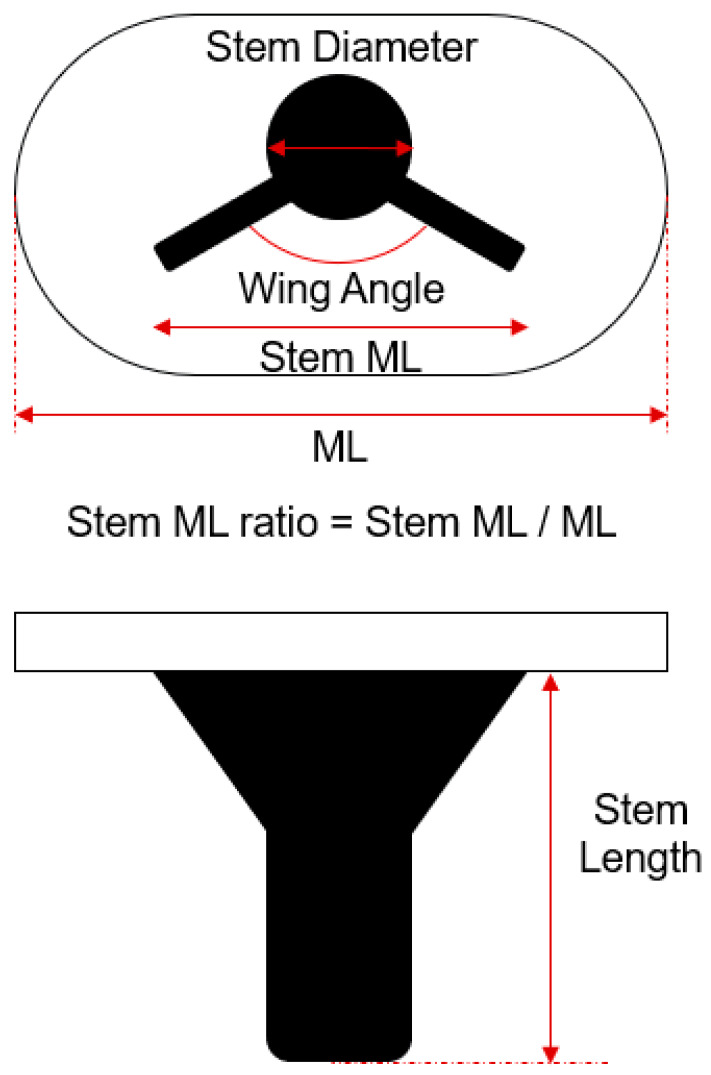
Definition of design parameter for the stem design of conventional TKA implants.

In this study, the following design parameters in Figure 2 were defined for tibia stem design, and each design parameter of commercial TKA implant products (Scorpio, Triathlon, Sigma, Attune, Vanguard, Genesis 2, Advance, Nexgen, Persona) were analyzed. From analysis, generic model that has four parameters was created: stem length, stem diameter, wing angle and stem M/L (Mediolateral) ratio. Anterior wing concept added as a new parameter as three levels (none, half, full).

### 2.2. Intact Model

Three-dimensional (3D) geometry of a linear finite element (FE) model of the knee was constructed based on the computed tomography (CT) images of a 62-year-old Asian female. The contours of the tibia were reconstructed from CT images using the commercially available software Mimics 17.0 (Materialise Ltd., Leuven, Belgium). CT images were obtained using a 64-channel CT scanner (Somatom Sensation 64; Siemens Healthcare, Erlangen, Germany). CT was performed with 0.1-mm slice thicknesses. The segmentation of the bone was performed using 3D adaptive thresholding. The study protocol was approved by our institutional review board.

### 2.3. Material Properties

Material properties of Cortical bone, Cancellous bone and tibial tray and were used from literature. Though we use CoCr for tibial tray, use of titanium will have a same tendency of result cause our FE model is linearly elastic. The material properties used in this study are listed in Table 1.

### 2.4. Loading & Boundary Conditions

**Figure 3:**
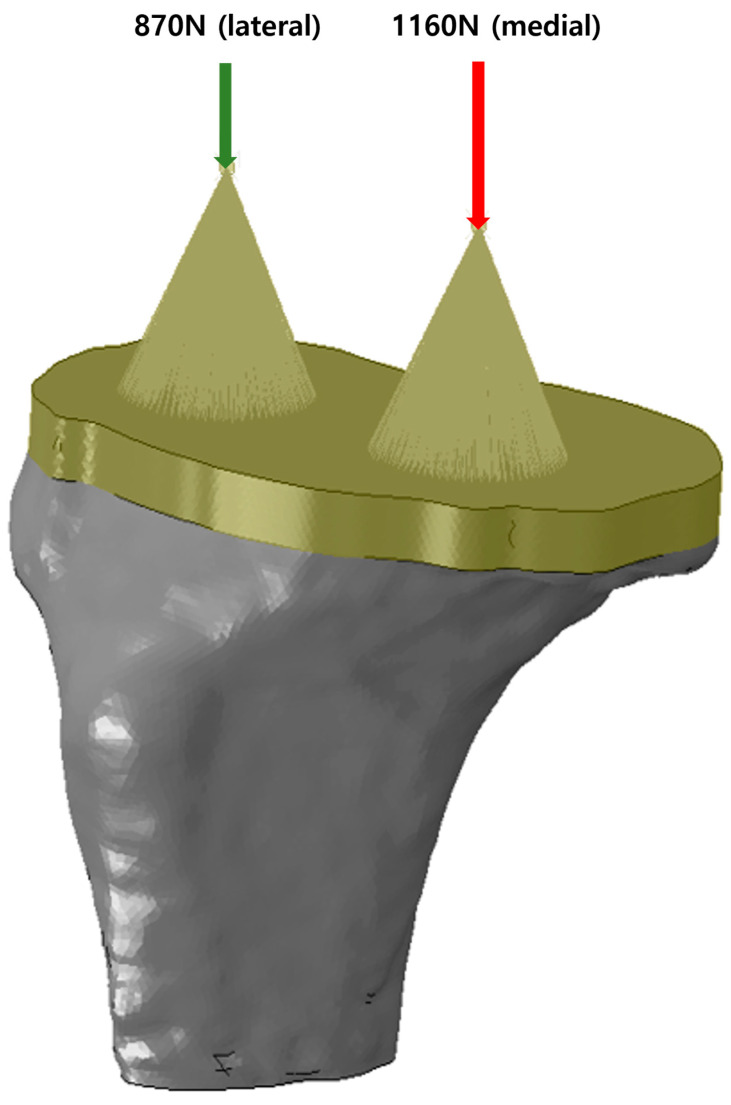
Loading conditions in the study.

In this study, a compressive loading condition corresponding to clinical relevance was used to evaluate the effect of the three stem lengths on various bone defects. Each bearing forces (lateral: 870 N, medial: 1160 N) were applied, considering that three times the load was applied in the late stance phase when a 70 kg adult walks. The compressive load on the tibial base is shown in Figure 3. The distal end of the tibial bone is fully constrained [6].

### 2.5. Mesh Convergence

The convergence of the FE model was investigated to complete the FE modeling. Mesh convergence was defined as the maximum displacement on trabecular bone were within 95% of the pressure of the next two smaller mesh sizes [8]. These criteria were met by average mesh size of 0.5 mm on tibial stem region, 0.8 mm on stem-around cortical and cancellous bone region and 1.0 mm on other bone regions. Quadratic tetrahedral elements of type C3D10 was applied. The numbers of created finite elements were as follows: cortical bone, 87,315, cancellous bone, 154,772, and tibial component, 86,490.

### 2.6. Finite Element Model

**Figure 4:**
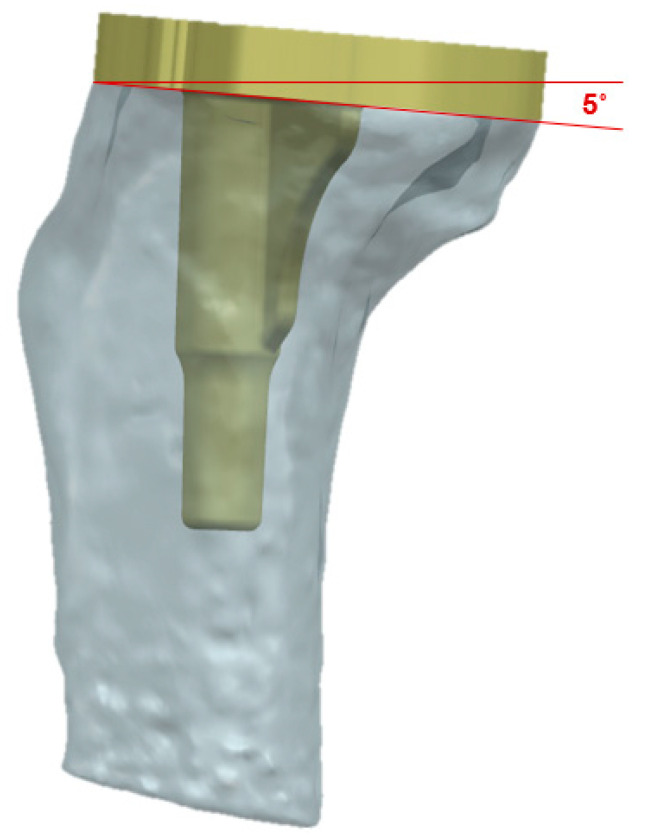
Tibia-component-implanted 3D model for TKA.

The bone models were imported into commercial CAD software (SP5.0, SolidWorks 2021, Dassault Systèmes, Vélizy-Villacoublay, France) and were appropriately positioned using surgical techniques. The FE model was generated using Hypermesh 11.0 (Altair Engineering, Inc., Troy, MI, USA). and analyzed using ABAQUS (version 6.11; Simulia, Providence, RI, USA), and the following surgical techniques were applied to the FE model. The stress and strain distributions on the trabecular bone depend on the position and shape of the cortical bone. The tibial axis was defined as the connection between the center of the tibial plateau and the center of the sphere that fits the talocrural joint. The proximal tibia was cut perpendicular to the tibial shaft with a posterior slope of 5°, and the cutting level was set at 8 mm from the highest side of the tibial plateau (all lateral condyles in this study, Figure 4). During implant insertion, the anteroposterior position was aligned with the anterior border, and the mediolateral position was positioned in the middle. The rotation of the tibial components was aligned to the line between the center of the posterior cruciate ligament footprint and the medial third of the tibial tubercle. For equivalent ML length of tibial plate, all conventional model’s ML length was changed to 68 mm (only for significant difference, change stem ML length either). The same insertion protocol was applied to all models.

### 2.7. Validation for FE Model

In this study, two loading conditions from previous studies were used to validate FE model. First, the walking loading conditions from Cho et al.’s study were applied to validate the FE model [6]. In the around stem tip area, the mean peak von Mises stress showed a difference within 10% compared to the control group. Second, the FE model was validated by applying stand-up loading conditions corresponding to activities of daily living for a 0° flexion angle of the knee [9]. The strain results from the FE analyses were compared with those obtained from mechanical tests. On average, the FE results showed a difference of within 7% compared to the experimental data. The values were highly accurate in most proximal tibia regions, while notable differences of approximately 10% were observed in the medial regions.

### 2.8. Design of Experiments (DOE)

Design parameters and level were determined in this study. Design of experiment was conducted using orthogonal arrays. We performed DOE 3 times to define the design parameters.

### 2.9. Criteria for Design Optimization

There can be two significant factors to evaluate performances of tibia stem, aseptic loosening and stress shielding. We classified each factor according to the short- and long-term effects as follows; aseptic loosening (short term) and stress shielding (long term). In a view of aseptic loosening, minimum principal stress was used as a criterion to follow literature. In a view of stress shielding, strain energy was used as a criterion to follow literature [10].

To optimize two responses with a trade-off relationship, we define multi-objective responses using weight and normalization. In addition, the weight factor of minimum principal stress, related aseptic loosening loosing, was 0.7 because we considered early failure more seriously [2,3,11,12]. The objective function in DOE 2 and DOE 3 for optimization was set as follows [13,14].

$$response=0.7*\frac{x_{1}-{PMiPS}_{min}}{{PMiPS}_{max}-{PMiPS}_{min}}+0.3\frac{x_{2}-{SE}_{min}}{{SE}_{max}-{SE}_{min}}$$

x_1_: The current value of minimum principal stress (PMiPS), which is associated with aseptic loosening.

x_2_: The current value of strain energy (SE), which is linked to stress shielding.

*PMiPS*_min_ and *PMiPS*_max_: Minimum and maximum values of the principal stress in the data set.

*SE*_min_ and *SE*_max_: Minimum and maximum values of the strain energy in the data set.

Finite element models were assessed of which conventional tibial plate and minimum principal stress and strain energy were calculated.

## 3. Results

### 3.1. Conventional Design Analysis

Using dimensions of Conventional TKA, we set boundaries in stem length, stem diameter, wing angle as shown in Table 2. We have created new stem model within these boundaries to ensure safety.

Comparison between base model and conventional tibia in minimum principal stress was conducted and base model was ranked 6 within 10 models in minimum principal stress rank. Comparison between base model and conventional tibia in strain energy was conducted and base model was ranked 2 within 10 models in strain energy rank. Therefore, we considered that our base model was competitive.

### 3.2. DOE 1—Screening

**Figure 5:**
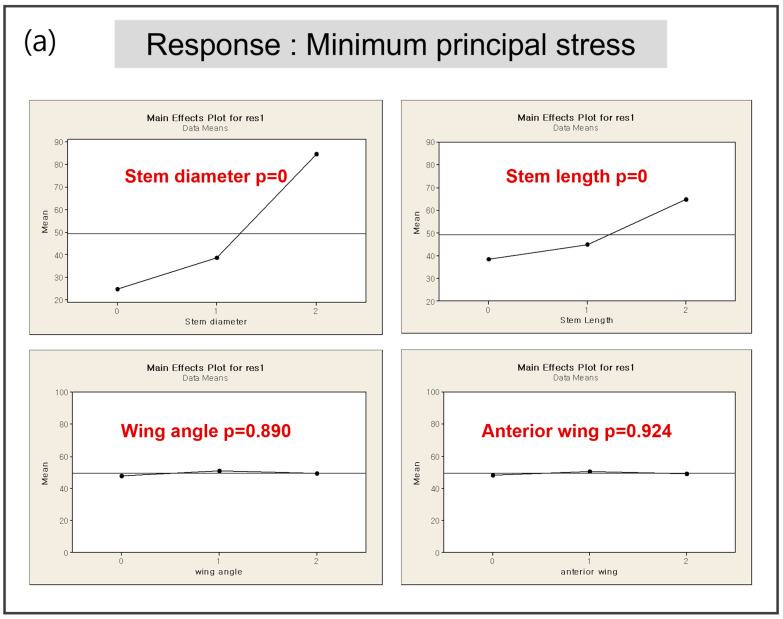

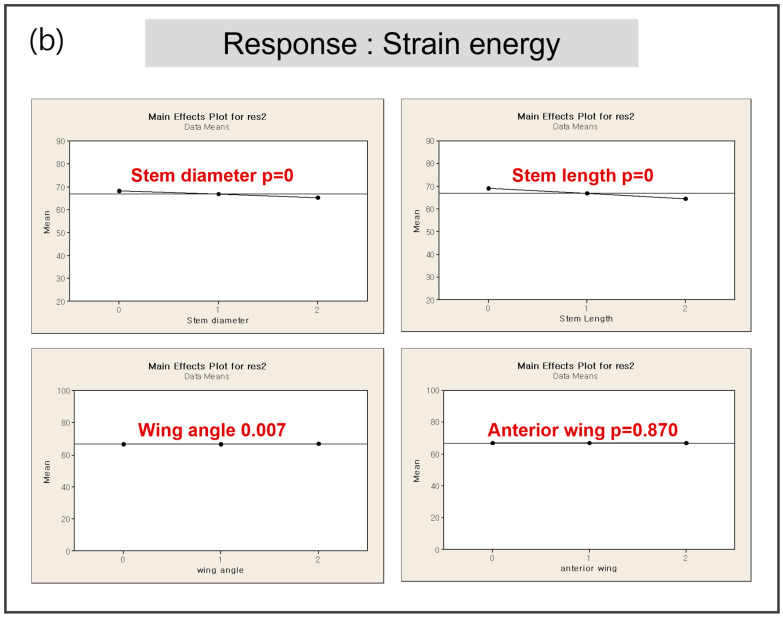
Response by design parameter in DOE1: (**a**) minimum principal stress (**b**) strain energy.

Design parameters and levels for DOE 1 were shown as Table 3. Stem diameter and stem length were dominant variables for each response. Wing angle has significant different only on strain energy. Therefore, wing angle was excluded for further DOE. There was trade-off relationship between two responses of dominant parameters (stem diameter, stem length, Figure 5) Because the anterior wing angle did not notably affect either strain energy or minimum principal stress, it was excluded from further DOE studies.

### 3.3. DOE 2—Stem Length and Stem Diameter

**Figure 6:**
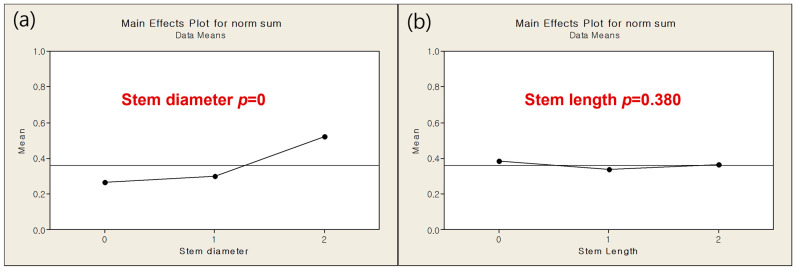
Multi-objective response by (**a**) by stem diameter (**b**) stem length.

In Figure 6, there was no significant effect of stem length on response that a value was determined in case of minimum responses and there was significant effect of stem diameter on response that we decided further DOE. Design parameters and levels for DOE 2 were shown as Table 4.

**Figure 7:**
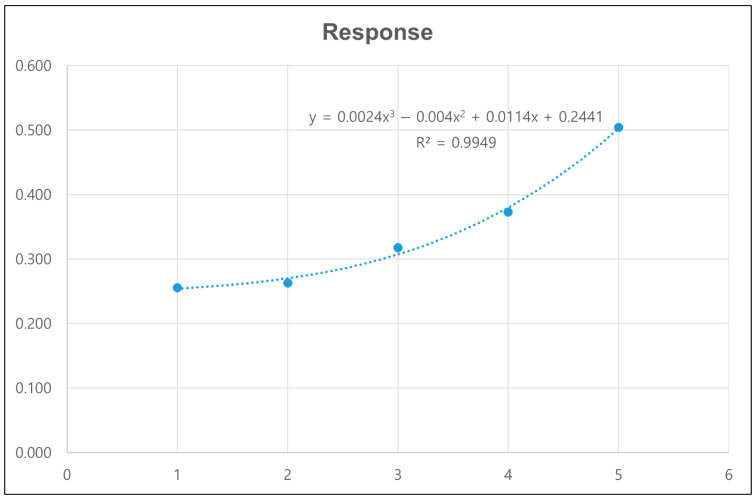
Regression curve for DOE 2.

In Figure 7, third order polynomial regression curve was calculated using RMSE approach (R-square = 0.9949). Regression curve validation was conducted in a point near optimum value. The difference between real and estimated value was 0%. The response of the optimized model with diameter 12 mm was 25.6% improve than the base model shown in Table 5. The multi-objective responses of base model and optimized model in DOE 2 were compared in Table 6.

### 3.4. DOE 3—M/L Ratio

In a view of bone preservation (minimal bone resection), further DOE was conducted. Design parameters and levels for DOE 3 were shown as Table 7. There were no significant effect of Stem length and M/L ratio on response. Stem length 40 mm and M/L ratio 0.61 was determined as a minimal bone resection. Analysis of variance for multi-objective using two parameters, stem length and M/L ratio, was conducted and each p-values of parameters were 0.857 and 0.723, respectively.

### 3.5. Final Model

Finally, design parameter was determined as follows: stem diameter 12 mm, stem length 40 mm; stem M/L ration 61% and stem wing angle 60°. Optimized model was analyzed using FEA. And optimized model has smaller M/L ratio, stem diameter which minimize bone resection. In addition, the final model had a low response, indicating that both of the two main factors, aseptic loosening and stress shielding effecting tibia stability of TKA, were satisfied to good performance. Below Table 8, Table 9 and Table 10 shows rank of M/L ratio, Stem diameter, Response.

### 3.6. Average Stress on Proximal Tibia

Figure 3 shows the average stress on the cortical and trabecular bones of the stem extension in the FE model according to the size of each medial tibial bone defect. The intact medial bone defect FE model with a longer stem extension showed that the average stress on the cortical and trabecular bones was small. All bone defect models of 2,4, 6, 8, and 10 mm, as well as the intact model.

## 4. Discussion

The most important finding of this study was that it could show the way for enhancing implant stability, reducing stress shielding, and minimizing the risk of aseptic loosening by the optimization of the tibial stem design in TKA. This study systematically investigated the effects of various design parameters using the DOE methodology to identify an optimized tibial stem configuration.

During knee movement, flexion and extension occur within the sagittal plane, accompanied by additional femoral external rotation and roll-back on the tibia during flexion [15,16,17]. These movements generate forces such as compression, tension, axial torque, varus/valgus moments, and shear, all of which must be resisted by the components of a TKA to ensure stability [18]. To mitigate these forces at the interface between the tibial component and the proximal tibia, projections like stems, pegs, or keels/wings can be integrated into the underside of the tibial component (Figure 1). These projections help reduce shear forces and axial displacement (lift-off) caused by varus-valgus moments [19]. Stems also limit micromotion at the bone/cement interface, thereby reducing the risk of aseptic loosening [20]. However, the presence of a stem introduces new shear forces between the stem and the proximal tibia. In this study, to implement the optimized model, four parameters were screened in DOE1 to measure their responses in terms of minimum principal stress and strain energy. The results from DOE 1 indicated that stem diameter and length are dominant factors influencing both the minimum principal stress and strain energy. The optimization process in DOE 2 showed that a smaller stem diameter (12 mm) combined with a stem length (40 mm) with the minimum amount of bone resection produced favorable outcomes in terms of both stress shielding and aseptic loosening. The regression analysis further validated these findings, demonstrating that the optimized model significantly outperforms the base model in multi-objective responses, with improvements in stress distribution and strain energy. As a key difference from previous studies [7], in DOE 2 there was no significant effect of stem length on response that a value was determined in case of minimum responses and there was significant effect of stem diameter on response that we decided further DOE. This is likely because, under the optimized model conditions, variations in stem diameter have a more significant impact on stress shielding and other related responses compared to changes in stem length.

Stems enhance the stiffness of the tibial construct and offer resistance to bending [21,22]. When tibial stems are sufficiently long, they engage the cortical bone as the metaphyseal flare tapers into the diaphysis. This engagement is more pronounced in press-fit stems, which are designed for bone ongrowth or ingrowth, compared to long stems with a smooth or polished surface. This configuration directs load directly from the stem to the cortical bone, causing stress to concentrate in this area and leading to stress shielding of the proximal metaphysis [22]. Even without direct cortical contact, the length of the stem correlates with the extent of stress shielding that occurs [23]. This stress shielding reduces bone density in the unloaded regions, increasing the risk of implant subsidence (tibial migration), loosening, and periprosthetic fractures. Another potential downside of longer stems is pain at the implant tip, where stress concentration happens [24]. In primary TKA with short-stem designs, load transfer and stress shielding are influenced by the implant’s geometry, material, tibial coverage, and the use of cement. Previous studies have predominantly focused on analyzing the impact of stem length on the outcomes of tibial constructs, as mentioned above. However, in this study, we developed a new response model by assigning different weight values to key factors influencing early and late failures after TKA. Specifically, minimum principal stress was identified as a critical factor in early failure, while strain energy was linked to late failure. This approach provided a new perspective, suggesting that in addition to stem length, stem diameter should also be considered when evaluating tibial geometry.

Contrary to initial expectations, the wing angle had a minimal impact on biomechanical responses, leading to its exclusion from further optimization stages. Similarly, the anterior wing design, inspired by the cross-shaped configuration, did not significantly reduce the minimum principal stress, indicating that its inclusion may not be necessary in the final design.

The DOE 3 analysis focused on minimizing bone resection by varying the M/L ratio and stem length. The findings revealed that there were no significant effect of stem length and M/L ratio on response. The final model, with a stem length of 40 mm and an M/L ratio of 0.61, was determined to achieve minimal bone resection while maintaining mechanical stability. This configuration effectively balances the need to preserve bone stock during implantation with the necessity of ensuring sufficient implant stability to prevent complications like aseptic loosening and stress shielding. The study found that reducing both the M/L ratio and stem length minimized bone resection without compromising the mechanical integrity of the implant, making it an ideal choice for enhancing long-term outcomes in TKA. Research on the M/L ratio of tibial stems in knee replacement surgery is somewhat limited, but it is a relevant factor in optimizing implant design and stability. The M/L ratio, which refers to the width of the stem relative to the width of the tibial component, plays a significant role in ensuring proper load distribution and reducing the risk of stress shielding or implant migration [7]. Several studies have touched on aspects of tibial stem design, including length, diameter, and surface finish, which all interact with the M/L ratio to influence the biomechanics of the implant. A balanced M/L ratio is important for maintaining stability, especially in cases where the tibial bone structure is compromised, such as in patients with osteoporosis or large bone defects. A well-chosen M/L ratio can help in achieving better alignment and fixation, thereby reducing the risks of loosening or subsidence [25]. While specific studies on the M/L ratio alone are scarce, related research indicates that this parameter, along with stem length and diameter, needs careful consideration during the design and selection of implants to ensure long-term success in TKA.

Additionally, while this study primarily focused on primary TKA, it is important to consider its implications for revision surgeries. Revision TKA presents unique challenges due to bone loss and the need for more robust fixation. The findings from this study, particularly regarding the significance of stem diameter in stress distribution, could inform future research aimed at optimizing tibial stems specifically for revision procedures. Further studies are warranted to evaluate how the optimized design might perform under the more demanding conditions of revision TKA, where additional fixation methods and stem modifications may be required to address the complexities of bone quality and implant stability.

This study also has several limitations. First, the exclusion of bone-cement interface from this study represents a significant limitation, as the interaction between bone and cement plays a critical role in the overall stability and long-term success of implants. Additionally, the TKA prosthesis was analyzed and implanted in a previously validated normal knee joint model derived from a 62-year-old Asian female. Including knee models representing end-stage osteoarthritis or other deformities in the finite element analysis could have provided insights into a broader range of clinical situations. The lack of consideration for this factor may lead to an incomplete understanding of the biomechanical behavior of the tibial stem, potentially affecting the generalizability of the findings to clinical settings where cemented fixation is commonly used [7]. Future research should incorporate bone-cement interference to provide a more comprehensive analysis of implant performance under realistic conditions. Second, significant limitation of this study is the use of isotropic, homogeneous, and linear material properties for cortical and cancellous bone. In reality, bone exhibits anisotropic and heterogeneous behavior, which significantly affects the mechanical response under various loading conditions [26]. The assumption of linear elasticity may oversimplify the complex nonlinear behavior of bone under physiological conditions, potentially leading to inaccuracies in stress and strain predictions. Third, the exclusive use of tetrahedral elements for meshing the entire model is another limitation. While tetrahedral elements provide flexibility in meshing complex geometries, they are less accurate in stress analysis compared to hexahedral elements, particularly in areas of high stress gradients [27,28]. This choice may lead to localized inaccuracies in the predicted stress and strain distributions. Employing a combination of hexahedral and tetrahedral elements, or conducting a detailed mesh convergence study, would enhance the accuracy and reliability of the FEA results. Fourth, this study does not include experimental or clinical validation of the FEA results, which is a critical limitation. Without validation, the accuracy of the predicted outcomes, such as stress shielding and aseptic loosening, remains uncertain [29]. The absence of validation may result in overestimation or underestimation of the model’s performance. Future work should incorporate validation through experimental testing or in vivo data to ensure the reliability of the findings and to bridge the gap between simulation and clinical application. Lastly, the most important aspect to consider in this study is the subjectivity of the weight setting. The weight factors (0.7 for minimum principal stress in early failure and 0.3 for strain energy in late failure) are determined based on the researcher’s subjective judgment of the importance of each factor. If the weight is not appropriately balanced, it may lead to suboptimal results in other criteria, such as long-term stress shielding. Additionally, trade-off management in response formula combines both objectives into one, which can oversimplify the relationship between the conflicting goals. For instance, improving one objective, such as reducing aseptic loosening, could inadvertently exacerbate another, such as increasing stress shielding. This interplay between objectives is not always accurately represented in a simple weighted approach. In conclusion, while the formula effectively captures the trade-off between early failure and long-term implant stability, the limitations regarding subjectivity in weighting and potential oversimplification of complex relationships should be carefully considered.

Future investigations should focus on understanding the effects of implant geometry on long-term outcomes such as bone remodeling and implant migration. Recent studies provide valuable insights into these areas. Giorgio et al. proposed an orthotropic continuum model with substructure evolution to explain bone remodeling mechanisms, aligning with Wolff’s law [30], while Allena and Rémond explored innovative approaches to medical treatments through mechanical interventions [31]. Integrating these perspectives into future analyses could significantly improve the understanding of long-term implant performance and their interaction with complex biomechanical environments.

## 5. Conclusions

This study aimed to optimize the tibial stem design for Total Knee Arthroplasty (TKA) using a systematic Design of Experiments (DOE) approach combined with finite element analysis (FEA). The results demonstrated that stem diameter and length are the most critical parameters influencing implant stability, stress shielding, and aseptic loosening. In contrast, wing angle and anterior wing design had minimal impact on biomechanical performance. The optimized tibial stem design, characterized by a stem diameter of 12 mm, stem length of 40 mm, mediolateral (M/L) ratio of 0.61, and wing angle of 60°, exhibited superior biomechanical stability and reduced stress shielding compared to conventional designs. The findings suggest that a carefully optimized tibial stem design can enhance implant longevity by improving load distribution while minimizing bone resection. Furthermore, this study provides new insights into the role of M/L ratio in tibial stem performance, an aspect that has not been extensively addressed in previous research.

This study contributes to the ongoing development of optimized tibial stem designs for TKA by providing a systematic methodology for balancing implant stability, stress shielding, and bone preservation. Future research should focus on experimental validation and patient-specific modeling to further refine tibial component design and improve clinical outcomes. Additionally, this research has the potential to be extended to the design of implants in other orthopedic applications.

## Figures and Tables

**Table 1:** Material properties.

	Material	Material Properties (MPa)	Poisson’s Ratio
Cancellous bone [6]	-	Loading moduli 449	0.3
Cortical bone [7]	-	Loading moduli 15,250	0.3
Tibial tray [6]	CoCr	Elastic modulus 210,000	0.3

**Table 2:** Dimensional boundaries for design parameters of tibia stem.

	Lower Limits	Upper Limits
Stem length (mm)	23.4	50.3
Stem diameter (mm)	11.5	19.8
Wing angle (°)	67.3	90
Stem ratio	0.53	0.73

**Table 3:** Design parameters and levels for DOE 1.

Parameter	Level			Explanatory
Stem diameter	12	14	Stem diameter	12
Stem length	40	45	Stem length	40
Wing angle	60	75	Wing angle	60
anterior wing	modeled	half	anterior wing	modeled
M/L ratio	0.68			Within conventional boundaries

**Table 4:** Design parameters and levels for DOE 2.

Parameter	Level					Explanatory
Stem diameter (mm)	12	13	14	15	16	Within conventional boundaries
Stem length (mm)	45					Minimum response
Wing angle (°)	60					Minimum response
Anterior wing	none					Minimum response
M/L ratio	0.68					Within conventional boundaries

**Table 5:** Validations results of DOE 2.

Diameter (mm)	Real Response	Estimated Response	Error
12.5	0.261	0.260	0%

**Table 6:** Comparison for the multi-objective response of base model and optimized model in DOE 2.

Multi-Objective Response	
Base Model	Optimized Model
0.33	0.256

**Table 7:** Design parameters and levels for DOE 3.

Parameter	Level	Explanatory
Stem diameter (mm)	12	Minimum response
Stem length (mm)	40	Minimum resection (no significant different)
Wing angle (°)	60	Minimum response
Anterior wing	none	Minimum response
M/L ratio	0.61	Minimum resection (no significant different)

**Table 8:** Verification of the final model: rank of M/L ratio.

Rank	Product	M/L Ratio
1	Persona	0.53
2	Sigma	0.55
3	Nexgen	0.56
4	Advance	0.58
5	Optimized model	0.61
6	Attune	0.63
7	genesis2	0.63
8	Vanguard	0.68
9	Scorpio	0.69
10	Triathlon	0.73

**Table 9:** Verification of the final model: rank of stem diameter.

Rank	Product	Stem Diameter (mm)
1	Scorpio	11.5
2	Optimized model	12
3	genesis2	12.2
4	Sigma	13.4
5	Persona	14.5
6	Advance	15.2
7	Nexgen	15.3
8	Attune	19.8

**Table 10:** Verification of the final model: rank of response.

Rank	Product	Response
1	Sigma	0.163
2	Optimized model	0.256
3	Advance	0.256
4	Attune	0.347
5	Vanguard	0.374
6	Genesis2	0.377
7	Scorpio	0.387
8	Triathlon	0.503
9	Nexgen	0.506
10	Persona	0.596

## Data Availability

Dataset available on request from the authors.

## Abbreviations

The following abbreviations are used in this manuscript:

TKA	Total Knee Arthroplasty
DOE	Design of Experiments
M/L	Mediolateral
3D	Three-dimensional
FE	Finite Element
CT	Computed Tomography
*PMiPS*	Minimum principal stress
SE	strain energy
*PMiPS*_min_	Minimum values of the principal stress
*PMiPS*_max_	Maximum values of the principal stress
*SE*_min_	Minimum values of the strain energy
*SE*_max_	Maximum values of the strain energy
